# STEMI Associated with Overuse of Energy Drinks

**DOI:** 10.1155/2015/537689

**Published:** 2015-02-19

**Authors:** Daniel Solomin, Stephen W. Borron, Susan H. Watts

**Affiliations:** Department of Emergency Medicine, Paul Foster School of Medicine, Texas Tech University Health Sciences Center at El Paso, El Paso, TX 79905, USA

## Abstract

Coronary artery disease (CAD) and ST-elevation myocardial infarction (STEMI) are predominantly diseases of middle-aged and older adults and when found in younger adults are usually associated with a strong family history. However, this report details the case of a nonobese 26-year-old Hispanic male who presented with an acute STEMI despite having no family history or other apparent risk factors for CAD or STEMI beyond a two pack-year smoking history and excessive energy drink consumption. The patient reported consuming between eight and ten 473 mL cans per day. Cardiac catheterization subsequently confirmed total occlusion of his left circumflex coronary artery.

## 1. Background

Energy drink consumption is a growing health concern due to limited regulation and increasing use, especially in younger demographics [1]. With substantially higher caffeine content than soft drinks or coffee beverages, in some cases, as well as other poorly studied substances, there is significant potential for harm, especially when consumed in large quantities. A review of energy drink toxicity cases in the National Poison Data System revealed moderate to major adverse effects in 15.2% of cases reported to regional poison centers, including seizures and dysrhythmias [2]. There have been two previous reports in the literature of STEMI associated with energy drink use by young people. Each patient was subsequently found to have normal coronary arteries. This report describes a novel case of a STEMI and established coronary artery disease in a young patient with no clear risk factors beyond a brief smoking history and excessive energy drink usage.

## 2. Case Presentation

**Figure 1:**
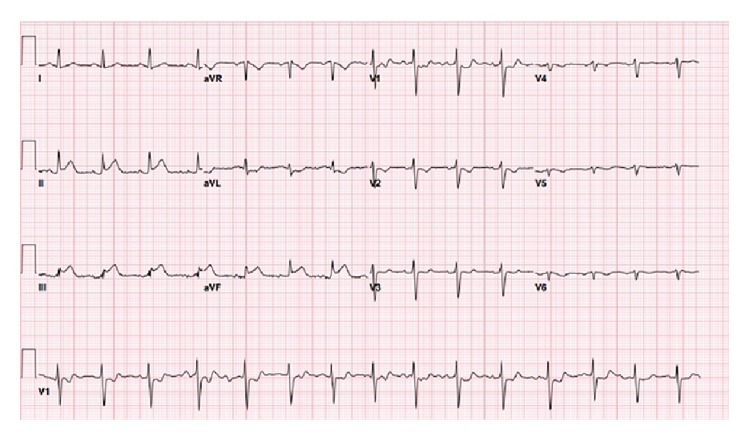
Initial electrocardiogram, showing evidence of acute inferior myocardial infarction.

The patient was a 26-year-old male who began having left-sided chest pain approximately 9 hours prior to presentation to the emergency department following drinking his usual quantity (~4L) of “Monster,” “Rock Star,” and other similar brands of energy drinks. The patient stated that he drank any kind of energy drink he could get access to: approximately eight to ten 473 mL drinks per day. The chest pain radiated to his left arm and his jaw but did not worsen with exertion. He also complained of numbness to his left arm, diaphoresis, nausea, and vomiting. His vital signs included a heart rate of 69, blood pressure of 132/73 mm/Hg, respiratory rate of 12, and oxygen saturation of 95% on room air. The patient was in significant distress and was diaphoretic, though not actively vomiting. The patient's heart showed regular rate and rhythm, lungs were clear to auscultation with no crackles, wheezes, or rhonchi, and the abdomen was soft and nontender without guarding or rigidity. His EKG showed significant ST-elevation in the inferior leads with reciprocal changes in the anterior leads (Figure 1). His initial troponin was 0.02 *μ*g/L (range 0–0.08 *μ*g/L) and CK-MB was 160.1 *μ*mol/L (range 0–266.9 *μ*mol/L); both tests were performed on a point-of-care testing device. The patient was taken to the cardiac catheterization lab before serial blood tests or further formal lab tests could be performed. The patient's serum caffeine concentration was not obtained, and the energy drink usage did not become known until after the patient had returned from the catheterization. The patient denied any illicit drug or stimulant use, and his urine toxicology screen was negative for any illicit drugs or stimulants, including common benzodiazepines, opiates, *δ*-9-tetrahydrocannabinol, amphetamines, and cocaine. The patient did however admit to smoking for the last two years, with approximately one pack (20 cigarettes) per day. The results of the patient's lipid profile were as follows: total cholesterol 5.65 *μ*mol/L (range 1.30–5.18 *μ*mol/L), HDL 0.96 (range 1.04–1.53 *μ*mol/L), LDL 2.69 *μ*mol/L (range <2.59 *μ*mol/L), and triglycerides 4.36 *μ*mol/L (range 0.40–1.70 *μ*mol/L). The patient had no history of diabetes, and each blood sugar check during his hospitalization was less than 6.11 *μ*mol/L (range 3.86–5.55 *μ*mol/L), but HbA1C was not checked. The patient's complete blood count and complete metabolic panel were within normal limits.

**Figure 2:**
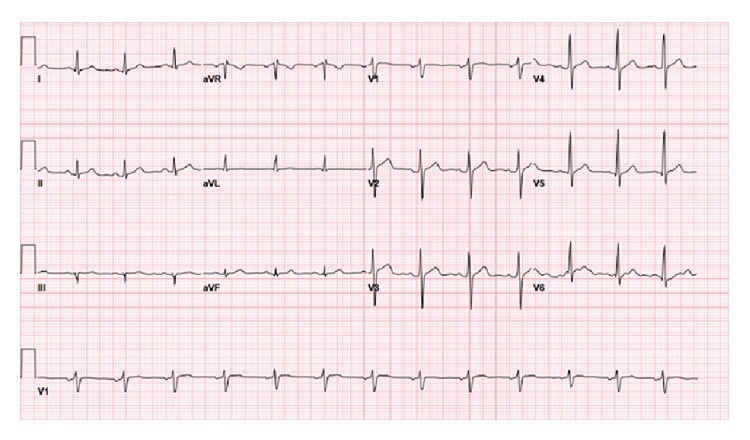
Electrocardiogram following cardiac catheterization and balloon angioplasty, showing resolution of inferior ST-elevation.

The patient underwent cardiac catheterization where a 100% occlusion of the left circumflex artery was observed, so a drug-eluting stent was placed after balloon angioplasty. The catheterization report is silent on the presence of any underlying coronary artery plaque in this region. The patient had “mild irregularities” in his left anterior descending coronary artery, in addition to the left circumflex artery occlusion, but no other coronary artery disease in his other coronary vessels was noted. His ST-elevation completely resolved after the catheterization (Figure 2). He remained in the hospital for two days after stent placement and experienced no further chest pain. He was discharged with prescriptions for an antiplatelet agent, an ACE inhibitor, a beta blocker, and a statin, and he agreed to stop smoking and consuming energy drinks.

## 3. Discussion

Energy drinks in various forms have existed for more than a century, including cocaine-containing Coca Cola which was introduced at the turn of the 20th century. However, energy drinks in their current incarnation are a fairly new phenomenon, with the sale of “Red Bull” beginning in 1997. There are multiple ingredients among the common brands of energy drinks, with the most frequent being caffeine, taurine, glucuronolactone, and ginseng. A typical energy drink has approximately 0.34 mg of caffeine per mL, meaning our patient consumed between 1.2 and 1.6 g of caffeine per day, with a lethal dose of caffeine being between approximately 10 g of oral caffeine based on animal studies [3]. However, smaller doses of caffeine may be fatal. Jantos and colleagues reported a fatal case in a 25-year-old woman who had ingested energy drinks containing 8.3 g of caffeine in association with ethanol [4]. There has been increasing concern about the safety profile of these beverages because there have been multiple case reports describing adverse effects from energy drink consumption, ranging from nausea/vomiting to palpitations and dysrhythmias. Consequently, legislation has been passed in several countries restricting the sale of energy drinks to minors, restricting the combination of these beverages with ethanol, and limiting the concentration of caffeine in these drinks. There appears to have been a decrease in suicides by caffeine following such legislation in Sweden [5].

A thorough literature review uncovered scant literature about lethal cardiac effects associated with energy drink consumption. One case of a lethal cardiac event was reported after ingestion of ecstasy (MDMA) in combination with energy drinks [6]. Two additional cases of STEMI after consumption of a large number of energy drinks have been reported in the literature. In one case, a 28-year-old male suffered cardiac arrest and ventricular fibrillation after consumption of ~7-8 cans of a “caffeinated energy drink.” Though no mention is made of the specific brand, the drink was said to contain both taurine and caffeine, similar to the drinks our patient regularly ingested [7]. There is a separate published case of a 19-year-old with chest pain after drinking 2-3 cans of Red Bull per day for the week prior to his presentation. Like our patient, he also had ST-elevation on his EKG [8]. However, in these published cases, both patients had normal coronary arteries on catheterization, unlike our patient. The authors of these cases postulated that the transient STEMI was possibly due to vasospasm caused by caffeine.

Caffeine acts primarily by competitively inhibiting adenosine receptors, but it also leads to increased catecholamine release. Though caffeine alone does not appear to have a significant effect on atherosclerosis [9], energy drinks contain multiple compounds in addition to caffeine, and there have been no definitive studies of these compounds to date, either alone or in the combinations found in energy drinks. However, one placebo-controlled physiological study has examined endothelial function and platelet aggregation among healthy medical students (20–24-year-old males) who consumed a single can (250 mL) of “a sugar-free energy drink.” This study reported an increase in platelet aggregation of ~14% (measured by optical aggregometry) compared to the controls, as well as a small decrease of the endothelial function [10].

We hypothesize that the significant quantity of energy drinks consumed by our patient, in the absence of any known genetic risk factors, contributed to the formation of the acute thrombus occluding the patient's coronary blood vessel. We hypothesize that vasospasm caused by excessive levels of caffeine, along with possible effects from other substances in energy drinks, reduced flow in the coronary vessel to such a degree that a thrombus was able to form. A reasonable alternative hypothesis is that of smoking related coronary artery vasospasm.

Further research into the topic of energy drink toxicity in general, as well as cardiac specific issues, is needed, and although evidence to date is scarce, it is probably prudent to recommend limited consumption of these drinks.
